# Supplementary material for: Development and validation of a risk prediction model of preterm birth for women with preterm labour symptoms (the QUIDS study): A prospective cohort study and individual participant data meta-analysis
Source: PLoS Med. 2021 Jul 6;18(7):e1003686. doi: 10.1371/journal.pmed.1003686 (PMC8259998; doi:10.1371/journal.pmed.1003686)
Supplement: S1 Text — Table A. Details of inclusion and exclusion criteria for QUIDS study. QUIDS study inclusion and exclusion criteria. fFN, fetal fibronectin; IPD, individual participant data; RCT, randomised control trial. Table B. Prespecified candidate predictors for inclusion in the QUIDS model and availability in each included study. Candidate predictors were prespecified, based on their potential to influence risk of preterm birth, and included: fFN concentration (ng/ml), previous spontaneous preterm birth, nulliparity (no previous pregnancy >24 weeks), gestation at fFN test (weeks), maternal age (years), ethnicity, body mass index (BMI kg/m2), smoking status, deprivation index, number of uterine contractions in set time period, cervical dilatation (cm), vaginal bleeding, previous cervical treatment for cervical intraepithelial neoplasia, cervical length (measured by transvaginal cervical length, mm), singleton or multiple pregnancy, and tocolysis. Only maternal age, BMI, ethnicity, smoking, nulliparity, multiple pregnancy, gestational age at assessment, previous spontaneous preterm birth before 34 weeks, cervical length, and fFN were available in each study, and, therefore, these 10 candidate predictors were included in the model development. Tocolysis was included in sensitivity analysis to explore any potential treatment effect on delaying birth. Table C. Details of eligible studies in the IPD meta-analysis. Six studies fulfilled the eligibility criteria; at the time, only one study was published (3), but three have subsequently been published (1, 2, 4). Five PIs agreed to provide data (Mol–Eufis; (2) van Baaren–Apostel-1; (1) Khalil–QFCAPS [Quantitative fetal fibronectin, Cervical length and ActimPartus for the prediction of Preterm birth in Symptomatic women]; unpublished, Shennan–EQUIPP [Evaluation of Fetal Fibronectin with a Quantitative Instrument for the Prediction of Preterm Birth]; (3) David–UCLH/Whit [University College London Hospital/Whittington], unpublished). The [file pmed.1003686.s002.docx]

**Supporting Information**

**Table of Contents**

| Table A Details of inclusion and exclusion criteria for QUIDS study | 3 |
| --- | --- |
| Table B Prespecified candidate predictors for inclusion in the QUIDS model and availability in each included study | 4 |
| Table C Details of eligible studies in the IPD meta-analysis | 5 |
| Table D Assessment of bias of studies included in the IPD meta-analysis | 6 |
| Table E Results two-stage random effects meta-analysis for heterogeneity of predictor effects | 7 |
| Table F Sites for prospective cohort study | 8 |
| Table G Description of fFN test and protocol for sampling | 10 |
| Table H Quality control measures for the Hologic Rapid fFN 10Q analyser | 11 |
| Table I Sensitivity analyses for model development | 12 |
| Table J Resource use items and unit collected via case report forms | 13 |
| Table K Model parameters used in the cost-effectiveness analysis | 14 |
| Table L Key assumptions for the cost-effectiveness models | 16 |
| Table M Baseline characteristics of the individual participant data meta-analysis dataset (A) and prospective cohort (B) study participants, derived from means of pooled imputations | 17 |
| Table N Probability of spontaneous preterm birth within seven days by gestational age in weeks | 18 |
| Table O Mean expected and observed (with 95% confidence interval [CI]) risk of spontaneous preterm birth within seven days for each of 10 risk groups (data for calibration plot [Figure 2]) | 20 |
| Table P Model performance measures for pre-specified sensitivity analyses of the QUIDS model | 21 |
| Table Q Resource use and cost estimates from QUIDS prospective cohort | 22 |
| Table R Cost-effectiveness results comparing the QUIDS risk predictor to a treat-all scenario and qualitative fFN (seven-day time horizon) | 23 |
| Table S Cost-effectiveness results comparing the QUIDS risk predictor to a treat-all scenario and qualitative fFN (lifetime horizon) | 24 |
| Fig A Decision tree framework | 25 |
| Fig B Net-Benefit curves comparing the QUIDS model with alternate models | 27 |
| Fig C Plot of receiver operator curve components for QUIDS risk prediction model | 28 |
| References | 29 |

**Table A Details of inclusion and exclusion criteria for QUIDS study**

| **Inclusion and exclusion for studies in IPD-meta-analysis** | - - 1. **Inclusion criteria**     2. Prospective cohort studies or RCTs of women with signs and symptom of preterm labour (as defined by investigators) that included quantitative fFN results determined by 10Q rapid fFN analyzer system and pregnancy outcome data; where the Principal Investigator was in agreement to collaborate and provide full data.     3. **Exclusion criteria**     4. Studies where fFN concentration was measured by ELISA and studies where IPD was not available for meta-analysis were excluded. |
| --- | --- |
| **Inclusion and exclusion for women in prospective cohort study** | ***Population and Eligibility***  Women with signs and symptoms of preterm labour at 22+0 to 34+6 weeks gestation in whom admission, transfer or treatment for preterm labour was being considered.  **Inclusion criteria at initial assessment:**   - 22^+0^ to 34^+6^ weeks (or earlier gestation if the fetus is considered potentially viable). - Signs and symptoms of pre-term labour including any or all of back pain, abdominal cramping, abdominal pain, light vaginal bleeding, vaginal pressure, uterine tightenings or contractions. - Hospital admission, interhospital transfer or treatment (antenatal steroids, tocolysis or magnesium sulphate) being considered due to signs of pre-term labour. - Aged 16 years or above.   **Additional inclusion criteria at speculum examination:**   - Cervical dilation ≤ 3cm - Intact membranes - No significant vaginal bleeding, as judged by the clinician   **Exclusion criteria:**   - Contraindication to vaginal examination (e.g. placenta praevia). - Higher order multiple pregnancy (triplets or more). - Moderate or severe vaginal bleeding. - Cervical dilatation greater than 3cm. - Confirmed rupture of membranes. - Sexual intercourse, vaginal examination or transvaginal ultrasound in the preceding 24 hours factors may invalidate results. These women will be initially excluded from the study, but can be included if still symptomatic after 24 hours, when fFN accuracy will be restored. |

QUIDS study inclusion and exclusion criteria. *RCT = randomised control trial. IPD = individual participant data.* *fFN = fetal fibronectin.*

**Table B Prespecified candidate predictors for inclusion in the QUIDS model and availability in each included study**

| **Candidate predictor** | **Apostel-1**  **Bruijn et al.** (1) | **Eufis**  **Bruijn et al.** **^(2)^** | **EQUIPP**  **Abbott et al.** **^(3)^** | **QFCAPS**  **Khalil et al.** | **UCLH/Whit**  **David et al.** |
| --- | --- | --- | --- | --- | --- |
| **Age** | **🗸** | **🗸** | **🗸** | **🗸** | **🗸** |
| **Body mass index (kg/m^2^)** | **🗸** | **🗸** | **🗸** | **🗸** | **🗸** |
| **Ethnicity** | **🗸** | **🗸** | **🗸** | **🗸** | **🗸** |
| **Smoking** | **🗸** | **🗸** | **🗸** | **🗸** | **🗸** |
| **Deprivation index** | **-** | **-** | **🗸** | **🗸** | **🗸** |
| **Nulliparity** | **🗸** | **🗸** | **🗸** | **🗸** | **🗸** |
| **Multiple pregnancy** | **🗸** | **🗸** | **🗸** | **🗸** | **🗸** |
| **Gestational age** | **🗸** | **🗸** | **🗸** | **🗸** | **🗸** |
| **Previous spontaneous**  **preterm birth < 34 weeks** | **🗸** | **🗸** | **🗸** | **🗸** | **🗸** |
| **Previous cervical treatment** | **🗸** | **-** | **🗸** | **🗸** | **🗸** |
| **Number of contractions** | **🗸** | **🗸** | **-** | - | - |
| **Vaginal bleeding** | **🗸** | **🗸** | **-** | - | - |
| **Cervical dilatation** | **🗸** | **🗸** | **-** | - | - |
| **Cervical length** | **🗸** | **🗸** | **🗸** | **🗸** | **🗸** |
| **Qualitative fetal fibronectin** | **🗸** | **🗸** | **🗸** | **🗸** | **🗸** |
| **Quantitative fetal fibronectin** | **🗸** | **🗸** | **🗸** | **🗸** | **🗸** |
| **Tocolysis** | **🗸** | **🗸** | **🗸** | **🗸** | **🗸** |

Candidate predictors were prespecified, based on their potential to influence risk of preterm birth, and included: fFN concentration (ng/ml), previous spontaneous preterm birth, nulliparity (no previous pregnancy > 24 weeks), gestation at fFN test (weeks), maternal age (years), ethnicity, body mass index (BMI; kg/m^2^), smoking status, deprivation index, number of uterine contractions in set time period, cervical dilatation (cm), vaginal bleeding, previous cervical treatment for cervical intraepithelial neoplasia, cervical length (measured by transvaginal cervical length, mm), singleton or multiple pregnancy and tocolysis. Only maternal age, BMI, ethnicity, smoking, nulliparity, multiple pregnancy, gestational age at assessment, previous spontaneous preterm birth before 34 weeks, cervical length, and fFN were available in each study and therefore these 10 candidate predictors were included in the model development. Tocolysis was included in sensitivity analysis to explore any potential treatment effect on delaying birth.

**Table C Details of eligible studies in the individual participant data meta-analysis**

| **INCLUDED** | | | | | | **EXCLUDED** |
| --- | --- | --- | --- | --- | --- | --- |
| **Study** | **Apostel-1**  **Bruijn et al.^. (1)^** | **Eufis**  **Bruijn et al.** (2) | **EQUIPP**  **Abbott et al.** **^(3)^** | **QFCAPS**  **Khalil et al.** | **UCLH/Whit**  **David et al.** | **STOP**  **Levine et al.** **^(4)^** |
| **Setting** | 10 Dutch hospitals | 10 European hospitals | 5 UK centres | 2 UK centres | 2 UK centres | 1 USA centre |
| **Dates** | 2009 - 2012 | 2012 - 2014 | 2010 - 2012 | 2012 - 2016 | 2009 - 2010 | 2013-2015 |
| **Inclusion criteria** |  |  |  |  |  |  |
| Signs/symptoms of preterm labour | - Contractions (>3/30min)  - Vaginal bleeding  - Abdominal/back pain | - Contractions (>3/30min)  - Vaginal bleeding  - Abdominal/back pain | - Contractions (>2/30min)  - Vaginal bleeding  - Abdominal/back pain | - Contractions (>4/20 min) | - Regular intermittent abdominal pain / uterine contractions every 10 min  - clinical suspicion of threatened preterm labour | - Preterm labor symptoms including vaginal/pelvic pressure, spotting, cramping, abdominal pain, or contractions |
| Intact membranes | **🗸** | **🗸** | **🗸** | **🗸** | **🗸** | **🗸** |
| Gestational age | 24 – 34 weeks | - 24 – 34 weeks | 22– 35 weeks | 24– 35 weeks | 22 – 35 weeks | 22-34 weeks |
| Singleton/multiple pregnancy | Singleton and multiple | Singleton and twins | Singleton and twins | Singleton only | Singleton and twins | Singleton only |
| Age | All | All | All | ≥ 16 years | All | All |
| **Exclusion criteria** |  |  |  |  |  |  |
| Cervical dilatation | > 3 cm | > 3 cm | > 3 cm | > 3 cm | > 3 cm | > 2 cm |
| Other | -Tocolytic treatment >12 hours  - Major fetal anomaly  - Suspected intrauterine infection  - Placental abruption  - Severe vaginal blood loss | -Tocolytic treatment >18 hours  - Major fetal anomaly  - Suspected intrauterine infection  - Placental abruption  - Severe vaginal blood loss |  | - Major fetal anomaly  - Antepartum haemorrhage - Mental health disorder  - Cervical cerclage | - Significant bleeding  - Sex intercourse /vaginal douche/ digital vag exam /transvaginal scan < 24hs of test | -Moderate-severe bleeding  -Major fetal anomaly  -Trauma that precipitated symptoms  - No birth information |
| ***Primary outcome*** | *Birth within 7 days of fFN test* | *Birth within 7 days of fFN test* | *Birth < 34 weeks gestation* | *Birth within 7 days of fFN test* | *Birth within 7 days of fFN test* | *Birth < 37 weeks gestation* |

Six studies fulfilled the eligibility criteria; at the time only one study was published, ^(3)^ but three have subsequently been published ^(1),^ ^(2),^ ^(4).^ Five PIs agreed to provide data (Mol - Eufis; ^(2)^ van Baaren – Apostel -1; ^(1)^ Khalil – QFCAPS [Quantitative fetal fibronectin, Cervical length and ActimPartus for the prediction of Preterm birth in Symptomatic women]; unpublished, Shennan - EQUIPP [Evaluation of Fetal Fibronectin with a Quantitative Instrument for the Prediction of Preterm Birth]; ^(3)^  David – UCLH/Whit [University College London Hospital/Whittington], unpublished). The PI of the 6^th^ study (STOP – Elovitz (4)) indicated data availability only after publication of the study, which occurred after completion of our analysis. *fFN = fetal fibronectin*

**Table D Assessment of bias of studies included in the individual participant data meta-analysis**

|  | **Apostel-1**  **Bruijn et al.^.^** ^(1)^ | **Eufis**  **Bruijn et al.** ^(2)^ | **EQUIPP**  **Abbott et al.** ^(3)^ | **QFCAPS**  **Khalil et al.** | **UCLH/Whit**  **David et al.** |
| --- | --- | --- | --- | --- | --- |
| ***Participant Selection*** |  |  |  |  |  |
| Was a consecutive or random sample of patients enrolled? | **🗸** | **🗸** | **🗸** | ? | ? |
| Was a case-control design avoided? | **🗸** | **🗸** | **🗸** | **🗸** | **🗸** |
| Did the study avoid inappropriate exclusions? | **🗸** | **🗸** | **🗸** | **🗸** | **🗸** |
| **Could the selection of patients have introduced bias?** | **Low risk** | **Low risk** | **Low risk** | **Unclear** | **Low risk** |
| ***Index Test*** |  |  |  |  |  |
| Were the index test results interpreted without knowledge of the reference standard? | **🗸** | **🗸** | **🗸** | **🗸** | **🗸** |
| If a threshold was used, was it pre-specified? | **🗸** | **🗸** | **🗸** | **🗸** | **🗸** |
| **Could the conduct or interpretation of the index test have introduced bias?** | **Low risk** | **Low risk** | **Low risk** | **Low risk** | **Low risk** |
| ***Reference Standard*** |  |  |  |  |  |
| Is the reference standard likely to correctly classify the target condition? | **🗸** | **🗸** | **🗸** | **🗸** | **🗸** |
| Were the reference standard results interpreted without knowledge of the results of the index test? | **🗸** | **🗸** | **🗸** | **🗸** | **🗸** |
| **Could the reference standard, its conduct, or its interpretation have introduced bias?** | **Low risk** | **Low risk** | **Low risk** | **Low risk** | **Low risk** |
| ***Flow and Timing*** |  |  |  |  |  |
| Was there an appropriate interval between index test(s) and reference standard? | **🗸** | **🗸** | **🗸** | **🗸** | **🗸** |
| Did all patients receive a reference standard? | **🗸** | **🗸** | **🗸** | **🗸** | **🗸** |
| Did all patients receive the same reference standard? | **🗸** | **🗸** | **🗸** | **🗸** | **🗸** |
| Were all patients included in the analysis? | **🗸** | **🗸** | **🗸** | **🗸** | **🗸** |
| **Could the patient flow have introduced bias?** | **Low risk** | **Low risk** | **Low risk** | **Low risk** | **Low risk** |
| ***Overall risk of bias*** | ***Low risk*** | ***Low risk*** | ***Low risk*** | ***Low risk*** | ***Low risk*** |

Assessment of bias of studies included in the individual participant data meta-analysis. *Risk of bias checklist adapted from (5)*

**Table E Results of two-stage random effects meta-analysis for heterogeneity of predictor effects**

|  | **Heterogeneity measures** | |
| --- | --- | --- |
|  | **T (95% CI)** | **I^2^ (95% CI) (%)** |
| Age | 0·0 (0·0 -0·03) | 0·0 (0·0 – 89) |
| Body Mass Index (kg/m^2^) | 0·0 (0·0 – 0·0) | 0·0 (0·0 – 26) |
| Smoking | 0·12 (0·0 – 2·4) | 9·6 (0·0 – 68) |
| Ethnicity |  |  |
| 1 White | - | - |
| 2 South Asian | 0·02 (0·0 – 6·4) | 0·57 (0·0 – 62) |
| 3 East Asian | 0·0 (0·0 – 0·0) | 0·0 (0·0 – 0·0) |
| 4 African, Caribbean, Middle-East | 0·0 (0·0 – 2·1) | 0·0 (0·0 – 68) |
| 5 Other | 0·0 (0·0 – 0·0) | 0·0 (0·0 – 0·0) |
| Nulliparity | 0·16 (0·0 – 1·2) | 25 (0·0 – 75) |
| Multiple pregnancy | 0·0 (0·0 -7·8) | 0·0 (0·0 – 94) |
| Gestational age | 0·0 (0·0 – 0·12) | 11 (0·0 – 91) |
| Previous spontaneous  preterm birth < 34 weeks | 0·58 (0·0 – 3·7) | 38 (0·0 – 80) |
| Cervical length | 0·0 (0·0 – 0·09) | 75 (0·0 – 98) |
| Quantitative fFN | 0·0 (0·0 – 0·0) | 26 (0·0 – 98) |

Results of two-stage random effects meta-analysis for heterogeneity of predictor effects individual participant meta-analysis. *fFN= fetal fibronectin*

**Table F QUIDS Prospective cohort study sites**

| **Name** | **Postcode** | **Prinicipal Investigator** | **Deliveries per annum** | **Neonatal Care Level** |
| --- | --- | --- | --- | --- |
| Bedford Hospital | MK42 9DJ | Mrs Sarah Reynolds | 2691 | Special Care Baby Unit |
| Birmingham City Hospital | B18 7QH | Dr Maheshwari Srinivasan | 5073 | Local Neonatal Unit |
| Birmingham Heartlands Hospital | B9 5SS | Dr Mani Malarselvi | 5535 | Neonatal Intensive Care Unit |
| Birmingham Women’s Hospital | B15 2TG | Dr R Katie Morris | 6770 | Neonatal Intensive Care Unit |
| Borders General Hospital | TD6 9BS | Dr Alex Viner | 966 | Local Neonatal Unit |
| Darlington Memorial Hospital | DL3 6HX | Dr Shilpi Mittal | 1787 | Special Care Baby Unit |
| Hinchingbrooke Hospital | PE29 6NT | Dr Sangeeta Pathak | 2108 | Local Neonatal Unit |
| King’s Mill Hospital | NG17 4JL | Dr Jyothi Rajeswary | 2815 | Local Neonatal Unit |
| Nevill Hall Hospital | NP7 7EG | Dr Anurag Pinto | 1763 | Special Care Baby Unit |
| Princess of Wales Hospital | CF31 1RQ | Mr Marsham Moselhi | *2000* | Local Neonatal Unit |
| Queen Alexandra Hospital | PO6 3LY | Mr Saumitra Sengupta | 5182 | Neonatal Intensive Care Unit |
| Queen Elizabeth Hospital, Gateshead | NE9 6SX | Mr Vaideha Deshpande | 1616 | Special Care Baby Unit |
| Queen Elizabeth University Hospital | G51 4TF | Dr Stewart Pringle | 5129 | Neonatal Intensive Care Unit |
| Queens Hospital Romford | RM7 0AG | Dr Chineze Otigbah | 7388 | Local Neonatal Unit |
| Royal Gwent Hospital | NP20 2UB | Dr Anurag Pinto | 3248 | Local Neonatal Unit |
| Royal Infirmary Edinburgh | EH16 4SA | Dr Shona Cowan | 6057 | Neonatal Intensive Care Unit |
| Royal London Hospital | E1 1BB | Mr Matthew Hogg | 4097 | Neonatal Intensive Care Unit |
| Singleton Hospital | SA2 8QA | Mr Marsham Moselhi | *2861* | Neonatal Intensive Care Unit |
| South Tyneside District Hospital | NE34 0PL | Mr Umo Esen | 1228 | Special Care Baby Unit |
| St George’s Hospital | SW17 0QT | Prof Asma Khalil | 4642 | Neonatal Intensive Care Unit |
| St Richards | PO19 6SE | Mr Attila Vecsei | 2454 | Local Neonatal Unit |
| St Thomas' Hospital | SE1 7EH | Prof Andy Shennan | 5541 | Neonatal Intensive Care Unit |
| Stoke Mandeville Hospital | HP21 8AL | Miss Aparna Reddy | *4950* | Local Neonatal Unit |
| University College London Hospital | NW1 2BU | Dr Davide Casagrandi | 5939 | Neonatal Intensive Care Unit |
| University Hospital of North Durham | DH1 5TW | Dr Shilpi Mittal | 2654 | Local Neonatal Unit |
| University Hospital of North Tees | TS19 8PE | Mr Steve Wild | 2699 | Neonatal Intensive Care Unit |
| Whipps Cross University Hospital | E11 1NR | Mr Matthew Hogg | 4292 | Special Care Baby Unit |
| Worthing Hospital | BN11 2DH | Mr Attila Vecsei | 2197 | Local Neonatal Unit |

UK sites included in QUIDS prospective cohort study **Table G Description of fetal fibronectin test and protocol for sampling**

| The Rapid fFN 10Q System (Hologic) measures the concentration of fFN (ng/ml or INVALID) in a vaginal swab sample  Samples for analysis were taken with a fFN specimen collection kit, which consists of a sterile polyester tipped swab and a specimen transport tube containing 1 ml extraction buffer (an aqueous solution containing protease inhibitors and protein preservatives including aprotinin, bovine serum albumin, and sodium azide). During speculum examination the sterile swab was lightly rotated across the posterior fornix of the vagina for ten seconds to absorb vaginal secretions. Samples were taken before any other swabs (e.g. for microbiology) or cervical manipulation and the speculum lubricated with normal saline as other lubricants may interfere with the antibody-antigen reaction of the test. Following specimen collection the swab was removed, immersed in extraction buffer, the shaft of the swab snapped off, and the transport tube sealed.  Before analysis samples were gently mixed and as much liquid as possible expressed from the swab by rolling the tip against the inside of the tube.  Vaginal swab samples were analysed by lateral flow; solid-phase immunochromatographic assay (the Rapid fFN Cassette), and interpreted in the 10Q Rapid analyser. 200 μL of the sample was pipetted into the sample application well of the Rapid fFN Cassette using a polypropylene or polyethylene pipette. The sample then flows from an absorbent pad across a nitrocellulose membrane via capillary action through a reaction zone containing murine monoclonal anti-fetal fibronectin antibody conjugated to blue microspheres (conjugate). The conjugate, embedded in the membrane, is mobilized by the flow of the sample. The sample then flows through a zone containing goat polyclonal antihuman fibronectin antibody that captures the fibronectin-conjugate complexes. The remaining sample flows through a zone containing goat polyclonal anti-mouse IgG antibody that captures unbound conjugate, resulting in a control line. After 10 minutes of reaction time, the intensities of the test line and control line are interpreted with the 10Q Rapid analyser and a printed result provided.  Within the QUIDS study fFN concentration was masked from clinicians (stored as a three-letter code). A POSITIVE/NEGATIVE/INVALID result was provided based on a single threshold of 50ng/ml. The result was invalid if the test did not meet internal quality controls that are performed automatically with every test. In the event of an invalid result, the test could be repeated with any remaining clinical specimen (usually sufficient for one or two repeats). |
| --- |

Description of fetal fibronectin test and protocol for sampling. fFN = fetal fibronectin

**Table H Quality control measures for the Hologic Rapid fFN 10Q analyser**

| The Hologic Rapid fFN 10Q analyser has integrated quality control measures. Use of a pre-calibrated reusable quality control cassette was used to verify that analyser performance was within specification. Quality control checks were mandatory, in that a test sample would not analysed if a quality control had not been performed in the preceding 24 hours.  Each fFN test also has an internal quality control.  All participating sites were requested to enrol in the Wales External Quality Assurance Scheme (WEQAS) Point of Care Quality Assurance Scheme which provided a sample for analysis to each site bimonthly, and provided confirmation of analyser performance and variability. Hologic offered training on sample collection and analysis to staff at sites participating in the study |
| --- |

Description of quality control measures for the fetal fibronectin test. fFN = fetal fibronectin

**Table I Sensitivity analyses for model development**

|  | **Model including all predictors** | | **Model including Cervical length**  **(3 studies)** | | **Model including Tocolysis** | |
| --- | --- | --- | --- | --- | --- | --- |
|  | ***Intercept*** | ***(95%CI)*** | ***Intercept*** | ***(95%CI)*** | ***Intercept*** | ***(95%CI)*** |
| Apostel-1  Bruijn et al. ^(1)^ | -7·849 | (-11·244 to  -4·454) | -1·805 | -5·33 to 1·41 | -6·983 | (- 8·827 to  -5·139) |
| Eufis  Bruijn et al· ^(2)^ | -8·529 | (-11·958 to  -5·100) | -2·408 | -6·00 to 0·77 | -7·609 | (-9·455 to  -5·763) |
| EQUIPP Abbott et al· ^(3)^ | -9·019 | (-12·444 to  -5·593) | - | - | -6·932 | (-8·553 to  -5·311) |
| QFCAPS  Khalil et al· | -8·700 | (-12·401 to  -4·998) | -3·114 | -7·26 to 0·49 | -7·078 | (-9·332 to  -4·824) |
| UCLH/Whit  David et al· | -9·324 | (-12·872 to  -5·777) | - | - | -7·441 | (-9·383 to  -5·499) |
|  | **Beta** | **OR (95% CI)** | **Beta** | **OR (95% CI)** | **Beta** | **OR (95% CI)** |
| qfFN | 2·033 | 7·64 (5·68 to 10·27)) | 0·006 | 1·01 (1·00 to 1·01) | 1·685 | 5·39 (3·98 to 7·30) |
| Age (year) | 0·024 | 1·02 (0·98 to 1·07) | 0·043 | 1·04 (1·00 to 1·10) | - | - |
| BMI (kg/m^2^) | 0·018 | 1·02 (0·96 to 1·08) | - | - | - | - |
| Smoking | -0·656 | 0·52 (0·24 to 1·13) | -0·991 | 0·37 (0·14 to 0·99) | -0·679 | 0·51  (0·24 to 1·09) |
| Ethnicity |  |  |  |  |  |  |
| 1 White | Ref |  | - | - | Ref |  |
| 2 South Asian | 1·066 | 2·90 (0·93 to 9·10) | - | - | 0·963 | 2·62  ((0·81 to 8·47) |
| 3 East Asian | -1·184 | 0·31 (0·04 to 2·49) | - | - | -1·137 | 0·32  (0·04 to 2·58) |
| 4 African, Caribbean, Middle-East | -0·216 | 0·81 (0·42 to 1·54) | - | - | -0·356 | 0·70  (0·37 to 1·34) |
| 5 Other | -0·252 | 0·78 (0·20 to 3·00) | - | - | -0·415 | 0·66  (0·17 to 2·61) |
| Nulliparity | 0·527 | 1·69 (1·06 to 2·71) | - | - | - | - |
| Multiple pregnancy | 0·852 | 2·34 (1·35 to 4·07) | - | - | 0·647 | 1·91  (1·11 to 3·28) |
| Previous spontaneous preterm birth < 34 weeks | 0·427 | 1·53 (0·78 to 3·03) | - | - | - | - |
| Gestational age at assessment (weeks) | 0·031 | 1·03 (0·96 to 1·11) | 0·092 | 1·10 (0·99 to 1·21) | - | - |
| Cervical Length (mm) | - | - | -2·941 | 0·05 (0·03 to 0·10) |  |  |
| Tocolysis | - | - |  |  | 1·856 | 6·40  (2·96 to 13·83) |
| ***Performance*** |  | |  | |  |  |
| Nagelkerke R^2^ | 0·21 | | 0·44 | | 0·43 | |
| AUC | 0·90 (95% CI 0·88 to 0·93) | | 0·92 (95% CI 0·89 to 0·94) | | 0·92 (95% CI 0·90 to 0·94) | |

Sensitivity analyses of different models developed using the IPD meta-analysis dataset. Multivariable logistic analysis of all candidate predictors; selected candidate predictors with cervical length (with data from the three studies with cervical length data completeness >80%); tocolysis. *qfFN = Quantitative fetal fibronectin ((qfFN+1)/100)^0·5). Cervical length = ((cervical length+1)/10)^0·5)*

**Table** **J Resource use items and unit collected via case report forms**

| The following resources items were collected in the cohort study: maternal admission, neonatal admissions, complications, transfers, treatments given. The equation below illustrates the main cost components in terms of the total mean cost per patient.  Per patient cost = C_Maternal admission_ + C_Neonatal admission_ + C_Complications_+ C_Transfers_ + C_Treatment_ | | | |
| --- | --- | --- | --- |
|  | Unit recorded in study | Unit cost | Source |
| Maternal admission^*^ | Hours (and minutes) | £449 per day | ^(6)^ |
| Corticosteroids^+^ |  |  |  |
| - Betamethasone | Per dose | £11.30 | ^(7)^ |
| - Dexamethasone | Per dose | £8.70 | ^(7)^ |
| Magnesium Sulphate^+^ | Hours (and minutes) | £7.70 | (7) |
| Tocolytics^+^ |  |  | (7) |
| - Nidedipine | Hours (and minutes) | £0.05 | (7) |
| - Indomethacin | Hours (and minutes) | £0.15 | (7) |
| - Glyceryl trinitrate | Hours (and minutes) | £0.02 | (7) |
| - Atosiban | Hours (and minutes) | £9.21 | (7) |
| - Other | Hours (and minutes) | £0.05 | (7) |
| Neonatal admission^*^ |  |  |  |
| - SCBU | Hours (and minutes) | £583 per day | ^(6)^ |
| - LNU | Hours (and minutes) | £920 per day | ^(6)^ |
| - NICU | Hours (and minutes) | £1,434 per day | ^(6)^ |
| Hospital transfer | Per transfer | £965 | ^(8)^ |
| Complications |  |  |  |
| - CPAP | Hours (and minutes) | £208 per day | (9) |
| - Intubation | Per treatment | £208 | (9) |
| - Oxygen | Hours (and minutes) | £18.90 per day | (9) |
| - Surfactant | Per treatment | £216 | (9) |
| - Surgery | Per treatment | £3,945 | ^(10)^ |

Details of resource use items and unit collected via case report forms. *^*^Hotel cost (58% of total stay cost) applied^. +^Dosage based on British National Formulary recommended standard dosage (mg). Unit cost is then estimated by multiplying dosage received in mg by unit cost per mg. C = cost. SCBU = Special Care Baby Unit. LNU= Local Neonatal Unit. NICU= Neonatal Intensive Care Unit. CPAP = Continuous Positive Airway Pressure therapy.*

**Table K Model parameters used in the cost-effectiveness analysis**

| **Treatment effect** | **Value** | **Standard Error** | **Probability distribution** | **Reference** |
| --- | --- | --- | --- | --- |
| Relative risk reduction of corticosteroids on mortality | 0.69 | 0.058 | Log-normal | (11) |
| Relative risk reduction of corticosteroids on morbidity | 0.66 | 0.036 | Log-normal | (11) |
| Neonatal outcomes | **Value** | **Standard Error** | **Probability distribution** | **Reference** |
| Probability of Death | 0.01 | 0.001 | Beta | (12) |
| Probability of major morbidity | 0.08 | 0.008 | Beta | (12) |
| Probability of minor morbidity | 0.38 | 0.038 | Beta | (12) |
| Probability of healthy | 0.53 | 0.053 | Beta | (12) |
| **QUIDS risk predictor performance at different risk thresholds** | **Sensitivity** | **Specificity** | **Probability distribution** | **Reference** |
| ≥2% risk | 0.79 | 0.84 | n/a | QUIDS study |
| ≥5% risk | 0.59 | 0.89 | n/a | QUIDS study |
| ≥10% risk | 0.49 | 0.90 | n/a | QUIDS study |
| ≥15% risk | 0.39 | 0.91 | n/a | QUIDS study |
| ≥20% risk | 0.22 | 0.92 | n/a | QUIDS study |
| ≥25% risk | 0.15 | 0.92 | n/a | QUIDS study |
| **Health utilities** | **Value** | **Standard Error** | **Probability distribution** | **Reference** |
| Healthy | 0.88 | 0.08 | Beta | ^(13)^ |
| Utility of minor morbidity | 0.83 | 0.21 | Beta | (14) |
| Utility of major morbidity | 0.76 | 0.23 | Beta | (14) |
| Utility of death | 0 | n/a | n/a | Assumption |

**Analysis**

The cost-effectiveness of alternative risk prediction strategies was evaluated by its incremental cost-effectiveness ratio (ICER), which was calculated according to:

ICER = ΔCosts/ΔQALY

Where ΔCosts is the difference in total costs between risk prediction strategies and ΔQALY is the difference in utility between risk prediction strategies. This incremental cost-effectiveness ratio can be compared against a societal willingness-to-pay for QALY gains (£20,000 in line with NICE reference case for cost per QALY. (15)) As considered QALYs a seven-day time horizon, we presented results in terms of cost per quality-adjusted life day (QALD), assuming a willingness-to-pay for QALD gained of £55 per day.

The cost-effectiveness of the risk prediction strategies could also be converted to the NMB as there are multiple comparators. The NMB is a measure of the health benefit, expressed in monetary terms, which incorporates the cost of the new strategy, the health gain obtained, and the societal willingness to pay for health gains (£20,000). The NMB is expressed using the following formula:

NMB = (E*WTP) – C

Where E = effectiveness; WTP = willingness-to-pay threshold; C = cost. The NMB approach is recommended when comparing more than one intervention and provides a clear decision rule (i.e. if NMB>0, the new strategy is cost-effective). Results can also be presented incrementally as the Incremental NMB.

**Table L Key assumptions for the cost-effectiveness models**

| The seven-day horizon base case analysis makes the following key assumptions:   - Clinicians always follow the test results (i.e. test results are never overruled). - Minor neonatal morbidity is captured as seven days of care in a lower level of neonatal care (SCBU). The cost of seven days in this type of care is applied and the health utility associated with this is based on the quality of life of an infant suffering from respiratory distress syndrome. - Major neonatal morbidity is captured as seven days of care in a high level of neonatal care (NICU). The cost of this is the NHS cost of this level of care for seven days and the health utility associated with this is based on the quality of life of an infant suffering from intraventricular haemorrhage (proxy for cerebral palsy). - The outcome of ‘did not deliver at seven days’ is attributed the same ‘full health’ QALYs as those babies delivered in ‘full health’. |
| --- |
| The lifetime horizon analysis makes the following key assumptions:   - Infants who are incorrectly not treated (i.e. false negatives), and experience major morbidity during the seven-day time horizon have lifetime cost and health implications. It is assumed that minor morbidity does not extend beyond seven days. - The quality of life for major morbidity is represented by the health utility associated with intraventricular haemorrhage (proxy for cerebral palsy). Lifetime costs associated with lifetime care for cerebral palsy women (£115,000 lifetime care) is incorporated. ^(8)^ Lifetime healthy utilities are determined based on an infant’s state in the model at seven days (dead, minor morbidity, major morbidity, healthy). These utilities ^(14)^ are extrapolated over an average lifespan and discounted to the present value. We do not capture the natural decreasing time profile of health utility over a lifetime since this is not known for infants with minor or major morbidity at seven days. |

Details of key assumptions made in the cost-effectiveness models. *QALY= Quality Adjusted Life Year*

**Table M Baseline characteristics of the individual participant data meta-analysis dataset (A) and prospective cohort (B) study participants, derived from means of pooled imputations**

| **Baseline characteristic** | **A. IPD meta-analysis dataset (model development)**  ***n* = 1,783** | **B. Prospective cohort study (external validation)**  ***n* = 2,924** |
| --- | --- | --- |
| Age years (mean [sd]) | 29·7 (5·6) | 28·2 (5·6) |
| BMI kg/m^2^ (median [IQR]) | 24·8 (22·0–28·4) | 25·4 (22·2–30·2) |
| Ethnicity (*n* [%]) |  |  |
| White | 1,206 (67·6%) | 2,578 (88·2%) |
| South Asian | 78 (4·4%) | 169 (5·7%) |
| East Asian | 46 (2·6%) | 8 (0·3%) |
| African, Caribbean, Middle East | 381 (21·4%) | 100 (3·4%) |
| Other | 72 (4·0%) | 69 (2·4%) |
| Current smoker (*n* [%]) | 208 (11·7%) | 614 (21·0%) |
| Nulliparous (*n* [%]) | 924 (51·8%) | 1,030 (35·2%) |
| Multiple pregnancy (*n* [%]) | 186 (10·4%) | 100 (3·5%) |
| Gestation (weeks; median [IQR]) | 29·4 (26·4–31·7) | 31·0 (27·9–33·1) |
| Previous spontaneous preterm birth < 34 weeks (*n* [%]) | 196 (11·0%) | 174 (6·0%) |
| **Outcome** |  |  |
| Spontaneous preterm birth within seven days | 139 (7·8%) | 85 (2·9%) |
| **fFN Results** |  |  |
| Qualitative fFN positive overall (*n* [%]) | 548 (30·7%) | 413 (14·1%) |
| Qualitative fFN positive in those with spontaneous preterm birth within seven days (*n/N* [%]) | 122/139 (87.8%) | 66/85 (77.6%) |
| Qualitative fFN negative in those with spontaneous preterm birth within seven days (*n/N* [%]) | 17/139 (12.2%) | 19/85 (22.4%) |
| Qualitative fFN positive in those without spontaneous preterm birth within seven days (*n/N* [%]) | 426/1,644 (25.9%) | 347/2,839 (12.2%) |
| Qualitative fFN negative in those without spontaneous preterm birth within seven days (*n/N* [%]) | 1,218/1,644 (74.1%) | 2,492/2,839 (87.8%) |
| Quantitative fFN overall (ng/mL; median [IQR]) | 11 (3–79) | 7 (4–22) |
| Quantitative fFN (ng/mL; median [IQR]) in those with spontaneous preterm birth within seven days (N = 139) | 391 (162-500) | 283 (73-500) |
| Quantitative fFN (ng/mL; median [IQR]) in those without spontaneous preterm birth within seven days (N = 1,644) | 9 (3-52) | 7 (4-20) |

Baseline characteristics of participants in QUIDS individual participant data meta-analysis dataset (A) and prospective cohort (B) study participants, derived from means of pooled imputations. fFN = fetal fibronectin. sd = standard deviation; IQR = interquartile range.

**Table N Probability of spontaneous preterm birth within seven days by gestational age in weeks**

| **Gestation (weeks)** | **N** | **Preterm Birth within 7 days** | **Preterm Birth Rate (%)** | **Lower CI** | **Upper CI** |
| --- | --- | --- | --- | --- | --- |
| 22 | 31 | 1 | 3.23 | 0.17 | 16.19 |
| 23 | 49 | 0 | 0 | 0 | 7.27 |
| 24 | 159 | 13 | 8.18 | 4.84 | 13.49 |
| 25 | 134 | 12 | 8.96 | 5.2 | 15 |
| 26 | 162 | 12 | 7.41 | 4.29 | 12.5 |
| 27 | 145 | 14 | 9.66 | 5.84 | 15.55 |
| 28 | 140 | 11 | 7.86 | 4.44 | 13.52 |
| 29 | 161 | 14 | 8.7 | 5.25 | 14.07 |
| 30 | 181 | 13 | 7.18 | 4.25 | 11.9 |
| 31 | 219 | 18 | 8.22 | 5.26 | 12.62 |
| 32 | 176 | 15 | 8.52 | 5.23 | 13.58 |
| 33 | 164 | 13 | 7.93 | 4.69 | 13.09 |
| 34 | 59 | 3 | 5.08 | 1.74 | 13.92 |
| 35 | 3 | 0 | 0 | 0 | 56.15 |

Probability of spontaneous preterm birth within seven days by gestational age in weeks

**Table O Data for calibration plot [Fig 2]**

| **Risk Group** | **Expected** | **Observed** | **Lower CI** | **Upper CI** |
| --- | --- | --- | --- | --- |
| 1 | 0.0042 | 0.0068 | 0.0000 | 0.0162 |
| 2 | 0.0065 | 0.0062 | 0.0000 | 0.0149 |
| 3 | 0.0077 | 0.0000 | 0.0000 | 0.0000 |
| 4 | 0.0084 | 0.0031 | 0.0000 | 0.0091 |
| 5 | 0.0095 | 0.0079 | 0.0000 | 0.0189 |
| 6 | 0.0109 | 0.0030 | 0.0000 | 0.0089 |
| 7 | 0.0121 | 0.0124 | 0.0000 | 0.0264 |
| 8 | 0.0145 | 0.0069 | 0.0000 | 0.0166 |
| 9 | 0.0219 | 0.0240 | 0.0062 | 0.0417 |
| 10 | 0.1367 | 0.2218 | 0.1679 | 0.2758 |

Mean expected and observed (with 95% confidence interval [CI]) risk of spontaneous preterm birth within seven days for each of 10 risk groups for calibration plot (data for calibration plot [Figure 2])

**Table P Model performance measures for pre-specified sensitivity analyses of the QUIDS model**

|  | *Primary analysis* | *Sensitivity analyses* | | |
| --- | --- | --- | --- | --- |
| **Performance Measure** |  | **Any preterm birth** | **Singletons only** | **Complete case** |
| ***Discrimination*** |  |  |  |  |
| AUC, C-statistic: point estimate | 0·89 | 0·88 | 0·90 | 0·89 |
| 95% CI | 0·85-0·93 | 0·83-0·92 | 0·85-0·94 | 0·85–0·94 |
| ***Calibration*** |  |  |  |  |
| Expected /Observed | 0·7839 | 0·7014 | 0·8111 | 0·775 |
| Calibration-in-the-large | 0·2884 | 0·4227 | 0·2418 | 0·303 |
| Calibration slope | 1·2041 | 1·1731 | 1·2747 | 1·211 |
| Re-calibration: intercept only |  |  |  |  |
| Intercept | -5·0637 | -4·9294 | -5·4849 | -5·423 |

Model performance measures for pre-specified sensitivity analyses of the QUIDS model: Any preterm birth (i.e. including provider initiated preterm births); singletons only and complete case analyses. *AUC = Area under the curve.*

**Table Q Resource use and cost estimates from QUIDS prospective cohort**

|  | **Resource use mean estimate in days (95% CIs), % of total receiving treatment** | | **Mean cost estimate per patient (95% CIs)** |
| --- | --- | --- | --- |
| Maternal observations (n) = 1,372 |  | |  |
| Maternal admission | 2.43 (1.99, 2.87) | | £611 (£501, £722) |
| Maternal hospital transfer | 6% (5%, 7%) | | £56 (£44, £67) |
| Corticosteroids | 37% (35%, 40%) | | £8.32 (£8, £9) |
| - Betamethasone | 75% (67%, 75%) | | £6 (£5, £6) |
| - Dexamethasone | 25% (21%, 29%) | | £2 (£1, £2) |
| Magnesium Sulphate | 0.34 (0.14, 0.54), 4% | | £4 (£2, £6) |
| Tocolytics | 0.42 (0.19, 0.67), 10% | | £0.04 (£0.02, £0.05) |
| - Nidedipine | 4.25 (1.70, 6.80), 91% | | £0.27 (£0.01, £0.04) |
| - Indomethacin | 0, 0% | | £0 |
| - Glyceryl trinitrate | 4.25 (1.70, 6.80), 6% | | £0.01 (£0.00, £0.01) |
| - Atosiban | 0, 0% | | £0 |
| Neonatal observations (n) = 735 |  |  |  |
| Neonatal admission | 11.56 (9.96, 13,17) | | £5,163 (£4,060, £6,265) |
| - SCBU | 5.68 (4.85, 6.52), 42% | | £1,848 (£1,578, £2,120) |
| - LNU | 1.70 (1.11, 2.28), 15% | | £870 (£570, £1,170) |
| - NICU | 2.02 (1.44, 2.60), 17% | | £2,900 (£2,069, £3,732) |
| Neonatal hospital transfer | 9% (7%, 11%) | | £103 (£78, £129) |
| Complications |  |  |  |
| - CPAP | 1.63 (0.10, 2.70), 22% | | £340 (£207, £472) |
| - Intubation | 0.4 (0.20, 0.59), 9% | | £83 (£43, £123) |
| - Oxygen | 1.91 (0.93, 2.89), 14% | | £40 (£19, £60) |
| - Surfactant | 7% (6% 9%), 5% | | £14 (£10, £19) |
| - Surgery | 1% (1%, 2%), 2% | | £75 (£36, £114) |

Resource use and cost estimates from QUIDS prospective cohort. *SCBU = Special Care Baby Unit. LNU= Local Neonatal Unit. NICU= Neonatal Intensive Care Unit. CPAP = Continuous Positive Airway Pressure therapy.*

**Table R Cost-effectiveness results comparing the QUIDS risk predictor to a treat-all scenario and qualitative fFN (seven-day time horizon)**

|  | Total  cost | Total QALDs | Incremental  QALDs | Incremental cost | ICER* (QALD) | Incremental NMB (QALD) |
| --- | --- | --- | --- | --- | --- | --- |
| Treat all | £1,182 | 6.149 |  |  |  |  |
| QUIDS  ≥2% risk | £316 | 6.148 | -0.0005 | -£866 | £1,732,000 | £856 |
| QUIDS  ≥5% risk | £267 | 6.148 | -0.0004 | -£49 | £122,500 | £41 |
| QUIDS  ≥10% risk | £249 | 6.147 | -0.0002 | -£18 | £90,000 | £14 |
| QUIDS  ≥15% risk | £239 | 6.147 | -0.0002 | -£10 | £50,000 | £6 |
| QUIDS  ≥20% risk | £234 | 6.147 | -0.0004 | -£5 | £12,500 | -£3 |
| QUIDS  ≥25% risk | £230 | 6.147 | -0.0002 | -£3 | £15,000 | -£1 |
| Qualitative fFN | £275 | 6.146 | -0.0007 | £44 | £-62,857 | -£58 |

Cost-effectiveness results comparing the QUIDS risk predictor to a treat-all scenario and qualitative fetal fibronectin (seven-day time horizon) *ICER is in the south-west quadrant in cost-effectiveness plane (cost saved per QALD lost). ICERs above £20,000 are considered cost-effective. QALD = Quality adjusted life days. ICER = Incremental cost-effectiveness ratio. NMB = Net monetary benefit. fFN = fetal fibonectin.

**Table S Cost-effectiveness results comparing the QUIDS risk predictor to a treat-all scenario and qualitative fFN (lifetime horizon**)

|  | Total  cost | Total QALYs | Incremental  QALYs | Incremental cost | ICER (QALY) | Incremental NMB (QALY) |
| --- | --- | --- | --- | --- | --- | --- |
| Treat all | £1,232 | 13.16 |  |  |  |  |
| QUIDS  ≥2% risk | £371 | 13.1593 | -0.0006 | £-840 | £1,400,000 | £827 |
| QUIDS  ≥5% risk | £328 | 13.1587 | -0.0006 | £-57 | £95,000 | £45 |
| QUIDS  ≥10% risk | £313 | 13.1584 | -0.0003 | £-7 | £23,333 | £2 |
| QUIDS  ≥15% risk | £305 | 13.1581 | -0.0003 | £3 | £-10,000 | £-10 |
| QUIDS  ≥20% risk | £304 | 13.1576 | -0.0005 | £2 | -£4,000 | £-12 |
| QUIDS  ≥25% risk | £303 | 13.1574 | -0.0002 | £-16 | £80,000 | £12 |
| Qualitative fFN | £331 | 13.1513 | -0.0061 | £35 | £-5,738 | £-157 |

Cost-effectiveness results comparing the QUIDS risk predictor to a treat-all scenario and qualitative fFN (lifetime horizon) *ICER is in the south-west quadrant in cost-effectiveness plane (cost saved per QALY lost). ICERs above £20,000 are considered cost-effective. QALY = Quality adjusted life years. ICER = Incremental cost-effectiveness ratio. NMB = Net monetary benefit. fFN = fetal fibonectin.

**Fig A Decision tree framework**


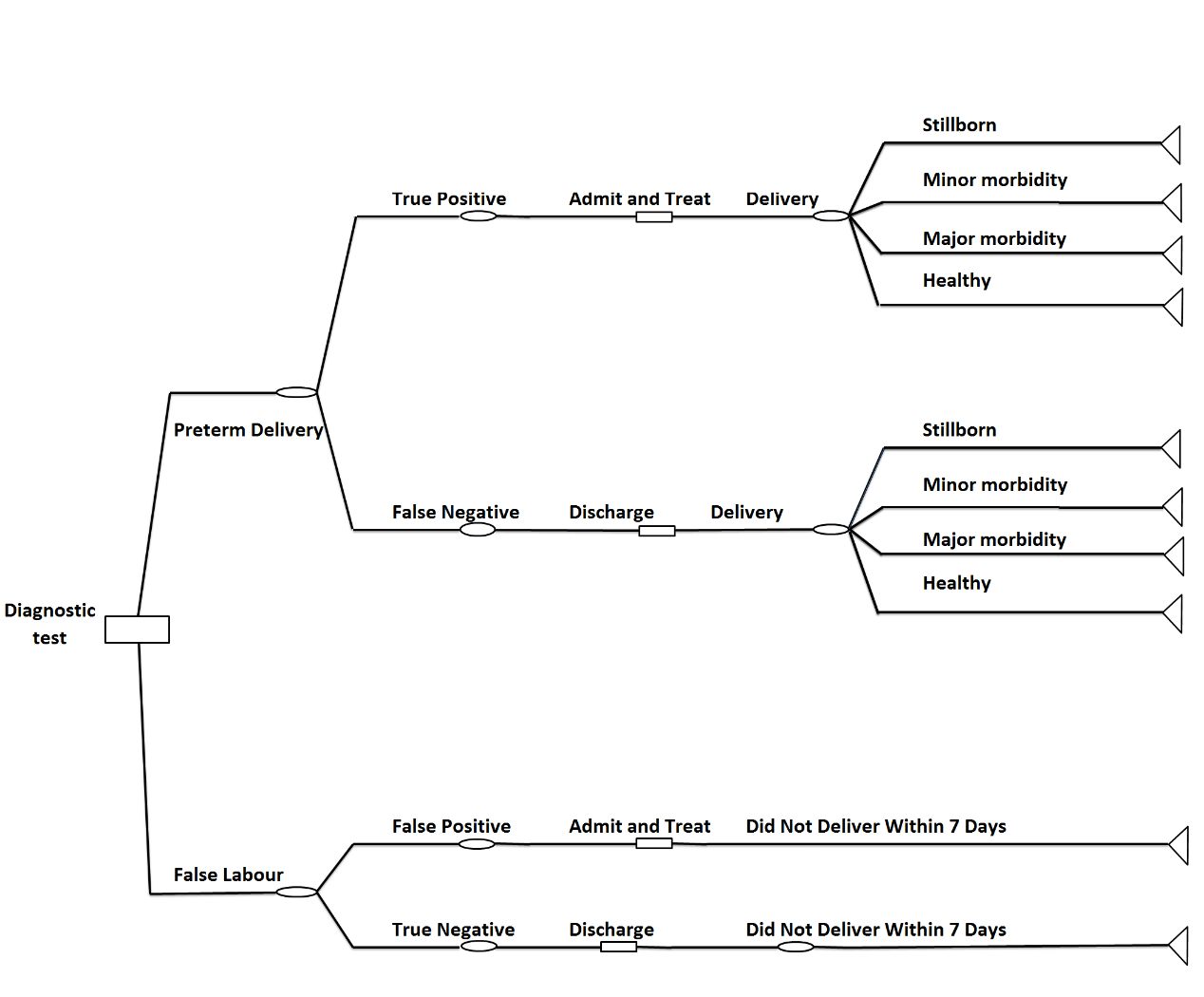


For each risk prediction strategy, the tree initiates with the true prevalence of preterm labour. Those identified as “high risk” by the strategy are admitted to a maternity unit with appropriate neonatal care facilities and receive antenatal corticosteroids. Those identified as “low risk” by the risk prediction strategy are not admitted and do not receive antenatal corticosteroids. The model can also be run under a hypothetical ‘treat all’ strategy where all participants are admitted. The final destination in the decision tree is one of five possible states for preterm births: stillborn, minor morbidity, major morbidity, full health and ‘did not give birth within seven days’.

Given this structure the model accounts for both the clinical and economic impact of false negative (low risk result, but gives birth within seven days) and false positive results (high risk result bit does not give birth within seven days) from the risk prediction strategies. False negatives represent missed opportunities to treat women with morbidity reducing antenatal corticosteroids, so infants born after a ‘false negative’ result have a greater probability of experiencing neonatal morbidity and mortality, incurring the associated costs, quality of life and survival impacts of these. False positives results will result in women being admitted to hospital unnecessarily, incurring additional and unnecessary cost of hospitalisation, inter-hospital transfer and treatment. It is assumed that there are no quality of life side-effects for receiving unnecessary treatment.

**Fig B Net-Benefit curves comparing the QUIDS model with alternate models**


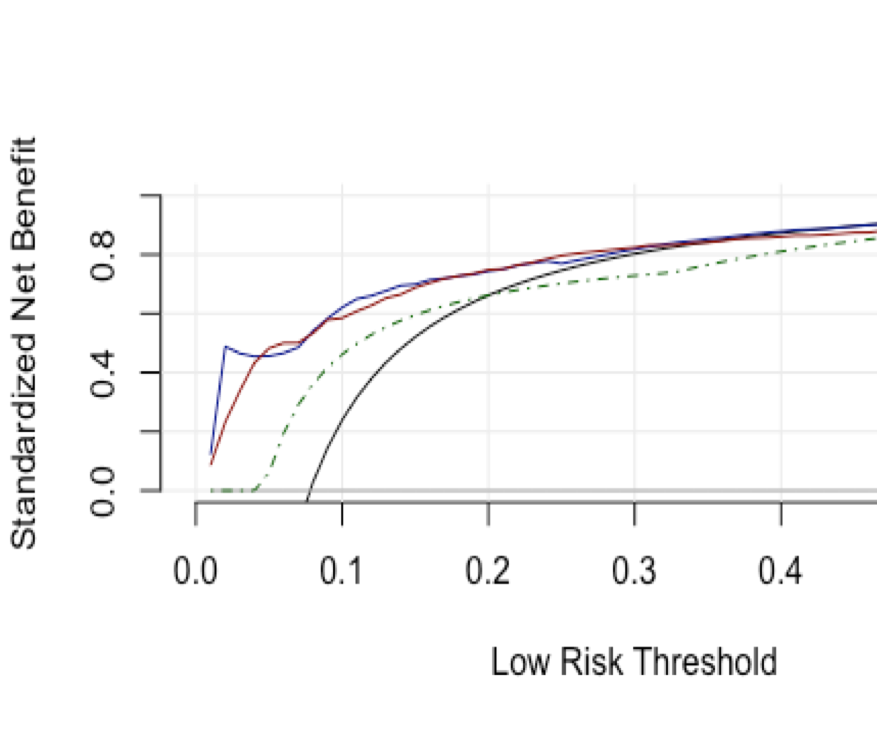


Net-benefit analysis. The figure shows the standardized net benefit (to visualize the potential benefit from reducing unnecessary treatment, and potential harms from ‘missing’ a case of preterm birth plotted on the same scale) at different % probabilities (from 0 – 60%) of spontaneous preterm birth within seven days as predicted by i) the QUIDS risk prediction model including clinical risk factors and quantitative fetal fibronectin (fFN; blue line); compared to a policy of treating all women with symptoms (dark grey line); an alternate model including clinical risk factors and quantitative fFN + cervical length (red line); an alternate model including cervical length alone (green dashed line); and a policy of treating no women (light grey horizontal line). Net benefit was calculated using the formula $\mathrm{sNB}^{\left( opt-out \right)}\left( r \right)=TNR\left( r \right)-\left( \frac{\rho}{1-\rho} \right)\left( \frac{1-r}{r} \right)\mathrm{FNR}\left( r \right)$ where standardized net benefit (sNB) is defined by True Negative Rate (TNR), the False Negative Rate (FNR), the prevalence of preterm birth within seven days (p), and the risk threshold (r) odds of low-risk designation at the % risk prediction. ^(16)^

**Fig C Plot of receiver operator curve components for QUIDS risk prediction model**


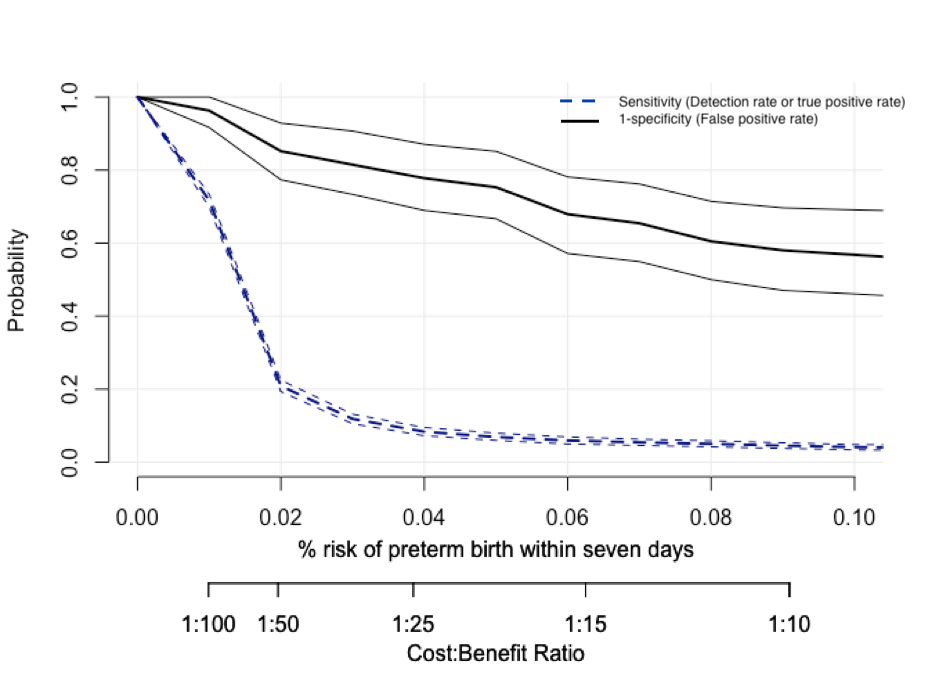


Sensitivity (detection rate or true positive rate; black line) and 1-specificity (false positive rate; blue dashed line) and 95% CI at different % probabilities (between 0 and 10%) of spontaneous preterm birth within seven days as predicted by the QUIDS risk prediction model. For example, at a predicted risk of 2%, the model has sensitivity of 0·85 (95% CI 0·76 to 0·93) and false positive rate of 0·28 (95% CI 0·27 to 0·30). The cost benefit axis is presented below the graph, with the cost indicating the ratio of ‘missed’ cases (cost) to the number of cases where unnecessary treatment was avoided (benefit), at different levels of risk predicted by the QUIDS model.

**References for S1 Text**

1. Bruijn M, Vis JY, Wilms FF, Oudijk MA, Kwee A, Porath MM, et al. Quantitative fetal fibronectin testing in combination with cervical length measurement in the prediction of spontaneous preterm delivery in symptomatic women. BJOG. 2016;123(12):1965-71.

2. Bruijn MM, Kamphuis EI, Hoesli IM, Martinez de Tejada B, Loccufier AR, Kuhnert M, et al. The predictive value of quantitative fibronectin testing in combination with cervical length measurement in symptomatic women. Am J Obstet Gynecol. 2016;215(6):793 e1- e8.

3. Abbott DH, NL. Seed, PT. Bennett, PR. Chandiramani, M. David, AL. Girling, J, Norman, JE. Stock, SJ. Tribe, RM. Shennan, AH. EQUIPP: Evaluation of Fetal Fibronectin with a novel bedside Quantitative Instrument for the Prediction of Preterm birth. Arch Dis Child Fetal Neonatal Ed. 2014;99:A150-A1.

4. Levine LD, Downes KL, Romero JA, Pappas H, Elovitz MA. Quantitative fetal fibronectin and cervical length in symptomatic women: results from a prospective blinded cohort study. J Matern Fetal Neonatal Med. 2018:1-9.

5. Whiting PF, Weswood ME, Rutjes AW, Reitsma JB, Bossuyt PN, Kleijnen J. Evaluation of QUADAS, a tool for the quality assessment of diagnostic accuracy studies. BMC Med Res Methodol. 2006;6:9.

6. Improvement N. National Schedule of Reference Costs 2017/18. 2018. Available at" <https://improvementnhsuk/resources/reference-costs/#rc1718> Accessed 29/4/2019

7. Committee JF. British National Formulary (online). London: BMJ Group and Pharmaceutical Press Available at: http://wwwmedicinescompletecom Accessed 29/4/2019

8. National Institute for Health and Care Excellence (NICE) Diagnostig Guidance 33. Biomarker tests to help diagnose preterm labour in woman with intact membranes. 2018. Available from: https://www.nice.org.uk/guidance/dg33/resources/biomarker-tests-to-help-diagnose-preterm-labour-in-women-with-intact-membranes-pdf-1053749042629 Accessed 29/05/2021.

9. Dani C, Ravasio R, Fioravanti L, Circelli M. Analysis of the cost-effectiveness of surfactant treatment (Curosurf(R)) in respiratory distress syndrome therapy in preterm infants: early treatment compared to late treatment. Ital J Pediatr. 2014;40:40.

10. NHS Scotland Information Services Division Maternity speciality costs 2018. Available at: <https://wwwisdscotlandorg/Health-Topics/Finance/Costs/Detailed-Tables/Speciality-Costs/> Accessed 29/4/2019

11. Roberts D, Brown J, Medley N, Dalziel SR. Antenatal corticosteroids for accelerating fetal lung maturation for women at risk of preterm birth. Cochrane Database Syst Rev. 2017;3:CD004454.

12. Manuck TA, Rice MM, Bailit JL, Grobman WA, Reddy UM, Wapner RJ, et al. Preterm neonatal morbidity and mortality by gestational age: a contemporary cohort. A J Obste Gynecol. 2016;215(1):103.e1-.e14.

13. Cooke RW. Health, lifestyle, and quality of life for young adults born very preterm. Archives of disease in childhood. 2004;89(3):201-6.

14. Carroll AE, Downs SM. Improving decision analyses: parent preferences (utility values) for pediatric health outcomes. The Journal of pediatrics. 2009;155(1):21-5, 5.e1-5.

15. NICE. Guide to the Methods of Technology Appraisal: 2013. 2013.

16. Brown M. Risk Model Decision Analysis 2018 Available at: <https://mdbrown.github.io/rmda/>. Accessed 29/4/2019
