# Supplementary material for: Development and validation of a risk prediction model of preterm birth for women with preterm labour symptoms (the QUIDS study): A prospective cohort study and individual participant data meta-analysis
Source: PLoS Med. 2021 Jul 6;18(7):e1003686. doi: 10.1371/journal.pmed.1003686 (PMC8259998; doi:10.1371/journal.pmed.1003686)
Supplement: Attachment — Submitted filename: QUIDS_response_v1.0.docx [file pmed.1003686.s003.docx]

Dr Sarah Stock

Wellcome Trust Clinical Career Development Fellow

Reader and Honorary Consultant Maternal and Fetal Medicine

Usher Institute, University of Edinburgh

NINE Edinburgh BioQuarter,

9 Little France Road,

Edinburgh EH16 4UX

sarah.stock@ed.ac.uk | +44 (0)7894629934

21^st^ April 2021

Dear Richard,

Many thanks for inviting us to submit a revised version of our manuscript "Development and validation of a risk prediction model of preterm birth for women with preterm labour symptoms (the QUIDS study): individual participant data meta-analysis and prospective cohort study." (PMEDICINE-D-21-01141R1) for consideration at PLOS Medicine. We thank the editor and reviewers for their constructive comments. We have addressed the specific points raised by the editors and reviewers below.

Requests from the editors:

- Please add "SJS is a member of PLOS Medicine's Editorial Board" to the competing interest statement.
- We suggest adapting the title to "...: A prospective cohort study and individual participant data meta-analysis".
- Please trim the "Background" subsection of your abstract.
- Please quote aggregate participant demographic details in the abstract.
- Please add a new final sentence to the "Methods and findings" subsection of your abstract, which should begin "Study limitations include ..." or similar and quote 2-3 of the study's main limitations.
- After the abstract, please add a new and accessible "author summary" section in non-identical prose. You may find it helpful to consult one or more published research papers in PLOS Medicine to get a sense of the preferred style.
- Please highlight any analyses that were not prespecified.
- In the Discussion section, we suggest expanding the discussion of study limitations.
- Please substitute "White" for "Caucasian".
- Please avoid claims such as "the largest", and if used add "to our knowledge".
- Generally the style should be "We included 10 predictors ...", for example, although numbers should be written out at the start of sentences.
- Throughout the paper, please adapt reference call-outs to the following style: "... every year [1,2]." (noting the absence of spaces within the square brackets).
- In the reference list, please abbreviate journal names consistently. Please convert all italics to plain text.
- Please break the attached TRIPOD checklist out into a separate file, labelled "S1_TRIPOD_Checklist" or similar and referred to as such in the Methods section.
- In the checklist, individual items should be referred to by section (e.g., "Methods") and paragraph numbers rather than by line or page numbers, as the latter generally change in the event of publication.
- **We have addressed all the Editorial Comments as suggested**

Comments from the reviewers:

*Reviewer #1:*

This study evaluated the value of quantitative fFN for prediction of imminent delivery among symptomatic women with threatened preterm labor. The authors proposed a new risk model and evaluated cost-effectiveness. Overall the study addresses an important problem and the methodology is sound.

1) It the introduction, the authors could perhaps mention studies aiming at developing signatures for prediction of preterm birth in symptomatic patients, such as <https://pubmed.ncbi.nlm.nih.gov/24828675/>and other.

**-See response to point 2 below.**

2) In the introduction, the authors state: "Transvaginal ultrasound measurement of cervical length is one strategy which has moderate predictive performance, but it requires specialised equipment and highly trained staff."

The authors could expand this sentence to add that there are personalized methods to assess cervical length which leads to better prediction including in symptomatic women (<https://pubmed.ncbi.nlm.nih.gov/32918893/>).

***-* We have not included these references as they are not directly relevant. We have restricted discussion to validated clinically used methods. Gene expression profiles are under development but not yet validated or used clinically. Personalised methods of cervical length have not been externally validated and are not widely used in the UK where this study was performed.**

3) Table 1 is said to show "Baseline characteristics of the IPD meta-analysis dataset (A) and prospective cohort (B) study participants, derived from means of pooled imputations". Superscripts could be used to specify for each variable how many cases were missing and hence needed imputation.

**- Levels of missing data are shown in Table S13 and Table S15, along with baseline characteristics and outcomes prior to imputation. We found that the levels of missing data was easier to see in these supplementary tables, and that addition of the necessary 15 superscripts complicated the main table and affected readability. However, we would be happy to add these in if the editors preferred.**

4) In Table 2 and line 440, smoking and Non-Caucasian Ethnicity seem have a protective effect against delivery within 7 days in symptomatic women. Perhaps these findings could be discussed in the context of what is known.

**-We have added the following lines to the discussion:**

**“Not all predictors in the QUIDS model performed as might have been predicted based on prior clinical knowledge. For example, smoking and not being of White ethnicity are both recognised to be risk factors for spontaneous preterm birth overall, but in our model, they were associated with a reduced risk of spontaneous preterm birth in women who present with symptoms. The reasons for this are unclear, but it may reflect an interaction between risk factors and management decisions. For example, clinicians’ perception of smokers as being at ‘high risk’ of preterm birth may mean they perform an fFN test more readily in the presence of minor symptoms in smokers than in non-smokers. It should be noted that unexpected findings are a recognised phenomenon in risk prediction models (Schuit E, Groenwold RH, Harrell FE Jr, de Kort WL, Kwee A, Mol BW et al. Unexpected predictor-outcome associations in clinical prediction research: causes and solutions. CMAJ. 2013 Jul 9;185(10):E499-505) As the main aim of a prediction model is to predict risk based on associations between predictors and the outcome, associations should not be interpreted causally and individual predictor effects are, in principle, not relevant. Nevertheless, model face validity is helped if predictor effects are as expected (Schuit E, Groenwold RH, Harrell FE Jr, de Kort WL, Kwee A, Mol BW et al. Unexpected predictor-outcome associations in clinical prediction research: causes and solutions. CMAJ. 2013 Jul 9;185(10):E499-505), and further work will explore how best to present our risk prediction model and supporting information to clinicians to optimise confidence in the model.”**

5) The authors state "Quantitative fFN was transformed (square root) because of non-linearity", yet the formula in Table 2 "((qfFN+1)/100)^-0·5)" shows an inverse square root (-0.5). This is unlike in line 441 where square root (0.5) is used instead. Please clarify which is the right one.

**-The ‘-‘ sign was in error. This has been removed**

6) "Candidate predictors (i.e. those, considered to have with potential to influence"

Remove "with"

**- We have removed “with”**

*Reviewer #2:*

"Development and validation of a risk prediction model of preterm birth for women with preterm labour symptoms (the QUIDS study): individual participant data meta-analysis and prospective cohort study" describes the development and validation of a model for predicting preterm birth (< 37 weeks) as its outcome, using vaginal fluid fetal fibronectin (quantitative fFN) as the primary novel risk factor/predictor of interest, together with nine other common predictors (reduced to five in total during development for the proposed QUIDS, formula in Line 440). Logistic regression with random effects meta-analysis was used as the underlying model, and compared to models including all potential predictors, and with cervical length, with all models returning similar performance. External validation and calibration was performed on a prospective cohort of over 2,900 patients, with consistent performance before and after calibration. A brief cost-effectiveness analysis supporting use of

QUIDS by assessing reduction in QALDs and monetary costs was provided, and suggests that usage of QUIDS at a suitable risk prediction threshold (2%; sensitivity 0.79, specificity 0.84) may be superior to existing treat-all or qualitative fFN-only approaches.

The study is generally meticulously documented, much of it in the accompanying supporting information. However, there remain some concerns that might be clarified:

1. The qualifying signs and symptoms of preterm labour (Line 163) applied in this study, might be described in greater detail.

**- We have clarified with the following text:**

*In the subsection* ***Model outcome, target population and candidate predictors:* “We included in the analyses pregnant women who were 22^+0^ to 34^+6^ weeks gestation with signs and symptoms of preterm labour (as defined by investigators), intact membranes and no contraindication to fFN testing (Table S1).”**

*In the subsection on* ***External validation:* “Women with signs and symptoms of preterm labour (any or all of back pain, abdominal cramping, abdominal pain, light vaginal bleeding, vaginal pressure, uterine tightenings or contractions), in whom…”**

2. It is notable that despite "gestational age at assessment" being one of the original ten candidate predictors, it was not judged sufficiently useful to be included in the final QUIDS model, despite patients' values for gestational age ranging from 26 to 32 weeks in the development dataset (Table 1). In particular, one might expect (premature) delivery at say 31 weeks, to be more common than at say 27 weeks, other conditions being equal. It might be discussed as to whether preterm births closer to 32 weeks are indeed more common from the available data, or if the presence of preterm labout symptoms overrides such expectations; the baseline probability of (preterm) delivery from gestational age might also be described.

**-The probability of preterm birth within seven days in women presenting with signs and symptoms of preterm labour was not influenced by gestation. The probability of spontaneous preterm birth within seven days by gestational age in weeks has been included as a supplementary table (S14) and referenced in the text.**

“**After backwards selection, predictors that remained in the QUIDS risk prediction model were quantitative fFN, smoking, ethnicity, nulliparity and multiple pregnancy (Table 2). Of note, gestational age did not influence the probability of spontaneous preterm birth within seven days (Table S14).”**

3. While a main focus of the paper is the inclusion of qfFN with other clinical predictors (with models involving all predictors/cervical length also examined), it does not appear to be established that the proposed QUIDS also significantly outpeforms yet more-parsimonious models (in particular, qfFN alone, or say qfFN+multiple pregnancy+current smoker for top three highest predictor weights); the backwards selection procedure for selecting predictors from the candidate set might also be described in greater detail.

- **As recognised by the reviewer, our aim was to develop and validate a model to predict preterm birth in women with signs and symptoms of preterm labour. We performed prespecified analysis according to our published protocol. The goal was to end up with a model that best predicts this outcome, meaning no subsets or submodels were considered. That said, the final model contains, apart from quantitative fFN, predictors that are readily available including smoking, ethnicity, nulliparity and multiple pregnancy, which means, in our opinion, that further model reduction is not necessary. If some of the included predictors were for example invasive or costly, then submodels may have been considered and compared, but that was not the case in our situation. We have expanded the text on backwards selection as follows.**

**“We used backwards selection based on an Akaike’s information criterion and a p-value of < 0·15...”**

4. Given the focus on qfFN in the study, it might be considered to provide more information about the distribution of qfFN (quantitative, ng/ml) depending on outcome, perhaps as a forest plot. This might also be considered for the other QUIDS predictors.

**- See response to point 5 below**

5. For the dataset demographics (as provided in Table 1), a confusion matrix relating major predictor "Qualitative fFN positive" and outcome "Spontaneous preterm birth within seven days" might additionally be presented (i.e. for each dataset, these correspond to the four values "qfFN positive/preterm birth", "qfFN positive/no preterm birth", "qfFN negative/preterm birth", "qfFN negative/no preterm birth")

***-* In light of these suggestions, we have included in Table 1 i) a confusion matrix for Qualitative fFN relating to preterm birth within seven days and ii) distribution of Quantitative fFN depending on outcome.**

6. For Figure 2, the data corresponding to each grouped observation point might be described (possibly in supporting information) and labelled.

- **The data corresponding to each grouped observation point for Figure 2 have been included in Table S17 and referenced in the figure legend.**

7. For Figure 3, the various lines might be labelled as to their respective probabilities, for each category.

**- The lines have been labelled**

Minor issues:

8. In Line 125, it is asserted that "The most established biochemical test is vaginal fluid fetal Fibronectin". This assertion might be directly supported by citation(s) if possible.

**- Reference added**

9. In Line 367, "internally validation" -> "internal validation"

**-Amended**

Reviewer #3:

Thank you for letting me review this paper about a new prediction model for preterm birth in symptomatic women. This paper is about combining clinical risk factors with quantitative fetal fibronectin in a risk prediction model for women presenting with signs and symptoms of preterm birth. The study is well designed with a development dataset and a validation dataset and I congratulate the authors for a great achievement. I have a few comments and suggestions for the manuscript.

Overall comments

1) I understand that the common practice in the UK is to use the qualitative fibronectin test rather than cervical length. Though, since a majority of countries, at least in Europe, use cervical length screening for preterm birth in symptomatic women, I would have liked to see the comparison of the predictive accuracy not only in comparison to qualitative fibronectin but also to cervical length screening with the best cut-off to the specific population. From the data, I understand that cervical length was not measured in all studies in the development dataset but would it be possible to use the studies where cervical length was measured, include the absolute numbers of cervical length in mm and provide data also for the predictive accuracy of cervical length only both in the development and validation dataset (if cervical length is available in the validation dataset)? If so, it would be of interest to readers in countries using cervical length screening to be able to

compare current practice to the prediction model.

**- The cervical length model (using data from 3 studies where this data was available) is shown in Table S9 and included in a net benefit analysis (Figure S3). It is included in the model as a continuous variable (rather than used at a single threshold) as per recommendations for prognostic model development (Royston P, Moons KG, Altman DG, Vergouwe Y. Prognosis and prognostic research: Developing a prognostic model. BMJ, 2009;338:b604). There were not sufficient cervical length data in the prospective cohort study to externally validate the model (as cervical length is not routinely used in current practice in the UK).**

2) The comparison of the new model to the standard of care with "treat all" below 30 gw and according to positive FfN above 30 gw would proabably not be the routine in other countries where a more restrictive approach below 30 gw would be more common (for example with a normal cervical length where the woman would be re-assessed later on). Would this change the cost effectiveness analysis if comparing the new model to a more conservative approach?

**- The cost effectiveness analysis was carried out from the perspective of the UK NHS and PSS. We agree that it is likely that the cost effectiveness analysis would change in other settings. This has been highlighted in the discussion as follows:**

**“The cost-effectiveness analysis was carried out from the perspective of the UK NHS setting, and may not be generalizable to other settings.”**

Specific comments

METHODS

1) Line 155; more useful predictor of PTB - compared to what? Dichotomous test?

**- Compared to qualitative fFN. This has been clarified.**

2) Line 167: One of the included papers had an outcome of PTB<34 weeks, not days to delivery. Did the author provide data so that thus could be calculated?

**-Yes, individual participant data were provided allowing us to develop the model as described.**

3) Line 168: an extra "with" could be removed (have with potential...)

**-Removed**

4) Line 173; change was to were

**-Changed**

5) Line 280: When adding iatrogenic preterm birth in the outcome - what is the underlying rationale to why fibronectin would be increased before iatrogenic PTB? Please explain.

-**We included all preterm births (spontaneous and provider initiated) in a sensitivity analysis to test the robustness of the model, as there is recognised cross-over between the pathologies which leading to spontaneous preterm birth and those necessitating provider initiated preterm birth (eg Partap U, Sovio U, Smith GC. Fetal Growth and the Risk of Spontaneous Preterm Birth in a Prospective Cohort Study of Nulliparous Women. Am J Epidemiol. 2016 Jul 15;184(2):110-9.)**

6) Line 296-304: Did you also considered a machine learning based approach to allow for non-linear correlations in the data?

**-No**

7) Line 301: How did it become apparent that the incidence of PTB in your population was lower than expected? Did you investigate this specifically in the dataset and was that a planned analysis?

**-We prespecified monitoring the event rate throughout the study.**

RESULTS

8) Table 1; Do you have any explanation to why the incidence of PTB was smaller than expected in your cohort? How was the external validity of your population? Did you hold a record of women declining participation or was there any differences in the population of the included centers in your study compared to other UK centers?

***-*The population was from 26 sites in the UK from a variety of settings (urban/rural; different levels of neonatal care) representative of UK setting.**

**We did not hold records of women declining participation in all sites. A requirement of being a participating site in QUIDS was an agreement that study machines that masked fFN results were the only ones available in clinical area. This meant we could informally monitor numbers of non-participants through records held on qfFN machines of fFN tests performed outwith QUIDS. Numbers were generally low. Other studies have shown similarly low levels of preterm birth in 7 days in women presenting with signs and symptoms of preterm labour (eg Levine LD, Downes KL, Romero JA, Pappas H, Elovitz MA. Quantitative fetal fibronectin and cervical length *in symptomatic women: results from a prospective blinded cohort study. J Matern Fetal Neonatal Med. 2019 Nov;32(22):3792-3800).***

**It seems likely that although “threatened preterm labour” is common, birth within seven days is genuinely rare in this population. It is possible that the majority of women who do give birth preterm either present in established labour, or present with different signs (eg membrane rupture or bleeding).**

9) Why do you think BMI did not fall out as a covariate after backwards selection when it is a known risk factor for PTB? Could it have to do with large missing data for BMI?

**- BMI did fall out as a covariate after backward selection. In our data there was no relationship between BMI and the probability of preterm birth within seven days in women presenting with symptoms of preterm labour. Although there were relatively high levels of missing data for BMI in the development cohort, we used multiple imputation which has been shown to be a valid technique for dealing with missing data within logistic regression models, resulting in less bias than excluding all women with missing data (Sterne JA, White IR, Carlin JB, et al. Multiple imputation for missing data in epidemiological and clinical research: potential and pitfalls. BMJ. 2009; 338: b2393.) Furthermore, levels of missing data for BMI were low in the prospective cohort study, and complete case analyses (sensitivity analyses) were very similar to those using imputed data. We have added missing data as a limitation in the discussion.**

**“Another potential limitation is the amount of missing data for certain variables. Multiple imputation was used to address missing data, which has been shown to be a valid technique for dealing with missing data within logistic regression models, resulting in less bias than excluding all women with missing data [39].”**

10) Line 370: Here you state that the model with cervical length performed better with an AUC of 0.92 but had similar potential utility? Again, referring to the overall comment, it would be interesting, taking that many countries use cervical length, to compare this to FfN and also in the final model for validation if possible.

**- See response to comment 1.**

11) Lines 417-418: In this analysis you seem to have compared the new model to "treat all" or "treat none". I understood from earlier in the manuscript that "treat all" was only below 30 gw. Could you please explain?

**- We compared the new model to treat all or treat none in the net benefit analysis according to established methodology (Vickers AJ, Van Calster B, Steyerberg EW. Net benefit approaches to the evaluation of prediction models, molecular markers, and diagnostic tests. BMJ. 2016;352:i6.). Including these ‘extreme’ options of management allows the potential benefit of different risk predictors to be visualised. The cost effectiveness analysis included a comparison with current recommendations for UK practice (NICE. National Institute of Health and Care Excellence NG25: Preterm labour and birth. London, 2015) used to represent standard care.**

12) Lines 440-442: The prevalence of smoking was high both in the development and in the validation cohort. Is this representative for the UK population / other populations with women with symptoms of PTB?

***-*The prevalence in the QUIDS prospective cohort is slightly higher than in the general UK population - in 2018/19 approximately 15% of pregnant women in the UK smoked (https://www.scotpho.org.uk/behaviour/tobacco-use/data/maternal-smoking/)**

13) Cost effectiveness: How is it possible to understand the potential benefits of timely MgSO4, transfer to a tertiary center and timely corticosteroids compared to the potential harm of repeated corticosteroids / over-treatment? Is this an analysis that we can actually relate to in clinical practice? I find it difficult to assess. Also, here it is stated that it was compared to a "treat all" strategy (see question 11).

**- The cost of treatment (including transfer, magnesium sulphate and antenatal corticosteroids) are included in the clinical pathway used in the decision model (which was informed by clinical experts). The use of each testing strategy (treat none, treat all, treat at specified probability cut-offs (0-100%) of preterm labour based on the prognostic model) and associated test accuracy directs different proportions of women towards “admit and treat” or “do not admit and treat”, which then has implications for the proportion of women entering into the final model states (stillborn, early infant death, morbidity, and healthy). Costs and utility values are attributed to the various states in the model and then summed along each pathway so that the associated total cost and total Quality Adjusted Life Years (QALYs) are calculated for each final model state. In this way the model captures the cost implications and quality of life implications of different testing strategies. As noted in the manuscript, the model assumes that there are no quality of life decrements associated with unnecessary treatment, however the cost of this was included.  Given current evidence base the potential harm of over-treatment of corticosteroids was assumed to be minimal and is therefore not included. The details of this are provided on page 25 of Supplementary Material.**

DISCUSSION

If not possible to add a comparison to CL only and to validate a prediction model with CL instead of FfN, it would be nice to see a section in the discussion adding this possibility for countries where CL is well implemented already and available day and night.

**- As per previous comments, we did develop a model including cervical length that we present in Table S9. This has similar performance to the QUIDS model, and was not superior in net benefit analysis (Figure S3).**

Yours sincerely,

Dr Sarah Stock
