# Supplementary material for: Development and validation of a risk prediction model of preterm birth for women with preterm labour symptoms (the QUIDS study): A prospective cohort study and individual participant data meta-analysis
Source: PLoS Med. 2021 Jul 6;18(7):e1003686. doi: 10.1371/journal.pmed.1003686 (PMC8259998; doi:10.1371/journal.pmed.1003686)
Supplement: Attachment — Submitted filename: QUIDS_response_v2.0.docx [file pmed.1003686.s004.docx]

Dr Sarah Stock

Wellcome Trust Clinical Career Development Fellow

Reader and Honorary Consultant Maternal and Fetal Medicine

Usher Institute, University of Edinburgh

NINE Edinburgh BioQuarter,

9 Little France Road,

Edinburgh EH16 4UX

sarah.stock@ed.ac.uk | +44 (0)7894629934

21^st^ April 2021

Dear Louise,

Many thanks for indicating your willingness to publish a revised version of our manuscript "Development and validation of a risk prediction model of preterm birth for women with preterm labour symptoms (the QUIDS study): individual participant data meta-analysis and prospective cohort study." (PMEDICINE-D-21-01141R1) for consideration at PLOS Medicine. We thank the editors for their suggested amendments. We have addressed the specific points raised by the editors below.

Requests from Editors:

Data availability:

Thank you for making your data available without restrictions on access, in a public repository. Prior to publication of your manuscript, please do ensure to provide us with the DOI or accession number required in order for this to be accessed.

**-	The DOI for data availability is https://datashare.ed.ac.uk/handle/10283/3885**

**-	I have set an embargo on unrestricted access until 1st August 2021, but this will be removed on publication of the manuscript. Until then approval to download the files can be requested through the site.**

**-	I will add citation details to the datashare site on publication of the paper; at the moment the citation link is to the published protocol.**

Could you please also confirm whether the data included in your meta-analysis (including the unpublished data from Khalil et al and David et al) will be available in the same public repository?

**-	I confirm all data relating to the publication are included, including unpublished data from Khalil and David.**

Abstract Methods and Findings:

For the meta-analysis component of your study, please provide the years during which the prospective cohort studies analysed took place.

**-	These have been added.**

For the prospective cohort study component of your study, please provide the years during which the study took place.

**-	These have been added.**

Please begin your Abstract Conclusions with "In this study, we observed ..." or similar, to summarise the main findings of your study.

**-	We have added “In this study, we found that a..”**

Thank you for providing an Author Summary. In the final sentence of ‘What do these findings mean?’, please also provide a bullet point summarising the main limitations of your study that will be accessible to a wide audience.

**-	We have added the following text.**

“Limitations were that there were low numbers of participants in the studies who were not White, and in there were some missing data in the risk predictor development cohort.”

Line 118 - please clarify what is meant by ‘ and the risk predictor generates’

**-	We have removed this text as agree it is not clear.**

Line 121 - please add the source of the data in each case (European / UK)

**-	European and UK added.**

Introduction:

Line 151 - please provide a couple of examples of the adverse maternal and neonatal effects

**-	The following text has been added, and reference 14 updated to better correspond to the new text**

“Unnecessary prophylactic treatments cause adverse maternal and neonatal effects, for example tocolytics can cause adverse drug reactions in mothers, and unnecessary antenatal corticosteroids are associated with adverse neurodevelopmental outcomes in children [12-14].”

Please indicate whether your study is novel, ensuring to temper assertions of primacy with ‘to the best of our knowledge’ or similar

Please remove the final line (indicating your study results and conclusion) from your Introduction.

**-	We have changed the last sentence to read**

“We aimed to develop and externally validate a new risk prediction model for preterm birth comprising these two elements using robust methodology”

Results:

Please incorporate Tables S13 and S15 into the main text of your results, as the baseline characteristics of study participants (without imputation)

**-	We have incorporated these tables showing the baseline characteristics of study participants (without imputation) as a single table within the main text. The table with means from imputed data is now Table S13. However, if the editors would like this moved back to the main text as an additional table, we would be happy to do this.**

Line 378 - Please provide the years during which the prospective cohort studies analysed took place.

**-	We have added the years 2009-2016**

Discussion:

Please re-organize the Discussion a little, as follows: a short, clear summary of the article's findings; what the study adds to existing research and where and why the results may differ from previous research; strengths and limitations of the study; implications and next steps for research, clinical practice, and/or public policy; one-paragraph conclusion.

**-	We have reorganized the discussion as suggested**

Line 519 - please revise the sentence beginning ‘ Further, to our knowledge the largest published study...‘ as the meaning is not entirely clear

**-	We have corrected this sentence to read**

“ Furthermore, to our knowledge, this is the largest published study of quantitative fFN..”

Figures and Tables:

Please provide titles and legends for each individual table and figure

Please define all abbreviations used in each figure in the respective figure legend e.g. Fig 1 “qfFN”

Please define all abbreviations used in each table in the respective table legend e.g. Table 1 “fFN”

-	**Title and legends have been provided with abbreviations defined.**

References:

Please ensure that journal name abbreviations match those found in the National Center for Biotechnology Information (NCBI) databases, and are appropriately formatted and capitalised.

Please remove additional information such as ‘[published Online First: 2018/11/06]’ in ref 1

Ref 15 - please provide a valid link as the text appears to have merged

**-	We have amended the references as advised.**

Supplementary files:

Please provide titles and legends for each individual table and figure in the Supporting Information. Please also define all abbreviations used in each table/figure in the respective table/figure legend.

-	**We have amended the supplementary files as requested. We have updated references for supplementary material.**

Thank you for providing the TRIPOD checklist; for points 21 and 22, please provide the (sub)sections within the manuscript text where this information can be found

**-	I have done this as far as is possible, but the data availability link and funder details are provided with submission not within the manuscript and so I presume will be published as metadata with the manuscript.**

Comments from Reviewers:

Reviewer #2: We thank the authors for addressing our previous points, and have no further comments.

**-	Many thanks**

Yours sincerely,

Dr Sarah Stock
