# Supplementary material for: Development and validation of a risk prediction model of preterm birth for women with preterm labour symptoms (the QUIDS study): A prospective cohort study and individual participant data meta-analysis
Source: PLoS Med. 2021 Jul 6;18(7):e1003686. doi: 10.1371/journal.pmed.1003686 (PMC8259998; doi:10.1371/journal.pmed.1003686)
Supplement: Attachment — Submitted filename: QUIDS_response_v3.0.docx [file pmed.1003686.s005.docx]

Dr Sarah Stock

Wellcome Trust Clinical Career Development Fellow

Reader and Honorary Consultant Maternal and Fetal Medicine

Usher Institute, University of Edinburgh

NINE Edinburgh BioQuarter,

9 Little France Road,

Edinburgh EH16 4UX

sarah.stock@ed.ac.uk | +44 (0)7894629934

6^th^ June 2021

Dear Louise,

Many thanks for indicating your willingness to publish a revised version of our manuscript "Development and validation of a risk prediction model of preterm birth for women with preterm labour symptoms (the QUIDS study): individual participant data meta-analysis and prospective cohort study." (PMEDICINE-D-21-01141R1) for consideration at PLOS Medicine. We have addressed the specific points raised below.

Requests from Editors:

*The manuscript looks almost ready for editorial acceptance, but I wondered if I could ask you please to correct Table S13 (the table seems to have been pasted twice) and to remove both instances of the word 'new' on lines 175 and line 520?*

- Table S13 has been corrected
- Both instances of the word 'new' on lines 175 and line 520 have been removed.

Yours sincerely,

Dr Sarah Stock
